# The Useful Medicinal Properties of the Root-Bark Extract of *Alstonia boonei* (Apocynaceae) May Be Connected to Antioxidant Activity

**DOI:** 10.1155/2014/741478

**Published:** 2014-01-27

**Authors:** Miracle Oluebubechukwu Obiagwu, Chibueze Peter Ihekwereme, Daniel Lotanna Ajaghaku, Festus Basden Chinedu Okoye

**Affiliations:** ^1^Department of Pharmaceutical and Medicinal Chemistry, Faculty of Pharmaceutical Sciences, Nnamdi Azikiwe University, PMB 5025, Awka, Anambra State, Nigeria; ^2^Department of Pharmacology and Toxicology, Faculty of Pharmaceutical Sciences, Nnamdi Azikiwe University, PMB 5025, Awka, Anambra State, Nigeria

## Abstract

Folkloric use of root-bark extract of *Alstonia boonei* in the treatment and management of many disease conditions may be associated with free radical scavenging as part of its mechanisms of action. We therefore evaluated the ability of different solvent fractions of the methanol extract, crude precipitate from the extract, and isolated compound from the crude precipitate for scavenging 2,2-diphenyl-1-picrylhydrazyl (DPPH) radical. Phytochemical analysis revealed the presence of useful phytocompounds. Ethyl acetate fraction showed better antioxidant activity with IC_50_ of 54.25 **μ**g/mL while acetone and methanol fractions have 121.79 and 141.67 **μ**g/mL, respectively. The crude precipitate and isolated compound showed IC_50_ values of 364.39 and 354.94 **μ**g/mL, respectively. The crude precipitate, fractions, and compound **1** showed antioxidant activity against DPPH radical although lower than that of ascorbic acid.

## 1. Introduction

Antioxidants are gaining special attention owing to their confirmed roles in prevention and management of chronic and degenerative diseases such as arthritis, cancer, cataract, and cardiovascular diseases [1]. *Alstonia boonei* is a large deciduous tree. It is widely distributed in Africa: Egypt, Cameroon, Central African Republic, Ghana, Code D'Ivoire, and Nigeria [2]. The various ethnomedicinal, chemical, pharmacological, and toxicological properties of *Alstonia boonei* were recently reviewed and the profile revealed that it is useful in the treatment and management of several illnesses [2]. The root bark is commonly used in West and Central Africa along with other herbs in the management of arthritis [3, 4]. The anti-inflammatory and antiarthritic properties of the root barks have been demonstrated [5, 6]. Furthermore, the antioxidant property of the stem bark has been documented [7]. Based on the diverse medicinal values of *A. boonei*, it is likely that inhibition of oxidative stress and free radicals might contribute to their medicinal effects. In this work, we hypothesize that the usefulness of the root extract in the management of chronic and degenerative diseases will likely have to be connected with the presence of some antioxidant property. Hence we set out to screen different solvent fractions of root-bark of *Alstonia boonei* for antioxidant effect since there is no literature report of antioxidant property in the root extract.

## 2. Materials and Methods

### 2.1. Plant Material

The root bark of *Alstonia boonei* was collected from Oba Nsukka Enugu State and authenticated by Mr. Alfred Ozioko of the Bio-resources Development and Conservation Programme Nsukka. The root bark was selected for this study because of scarce/limited scientific report on its medicinal activities compared to other parts of this plant. The root barks were cleaned, air-dried, and pulverized.

### 2.2. Extraction and Fractionation

The pulverized root bark (350 g) of *A. boonei* was cold-macerated with methanol and concentrated in vacuo to one-quarter of its volume using rotary evaporator. Semicrystalline precipitate from this concentrated extract was harvested and further purified by continuous washing with methanol. The remaining extract was adsorbed with silica gel and eluted in succession using ethyl acetate, acetone, and methanol.

### 2.3. Chromatographic Separation of the Crude Precipitate

For the column chromatographic separation, silica gel (60–200 mesh size) was used as the solid support and the column was developed with gradient mixtures of n-hexane : ethyl acetate (9 : 1, 4 : 1, 7 : 3, and 1 : 1) and ethyl acetate alone. Eluents were collected in 10 mL aliquots with small amber bottles. The fractions were monitored by TLC and bulked based on their pattern of separations into F1–F8. Compounds **1** and **2** were recrystallized from fractions 1 and 2, respectively.

### 2.4. Photochemical Analysis

This was done using standard methods described by Habourne [8] and Trease and Evans [9].

### 2.5. DPPH Test

The in vitro antioxidant activities of the crude precipitate, fractions, and compounds from the root bark were evaluated using the method of A. Patel and N. M. Patel [10]. DPPH solution (0.6 mmol) was freshly prepared using methanol as solvent; 0.5 mL of this solution was mixed with 0.5 mL of different dilutions (100, 200, 400, and 800 *μ*g/mL) of the crude precipitate, fractions, and compounds. The volume of the solution was adjusted with methanol to a final volume of 5 mL. After incubation in the dark for 30 minutes at room temperature, the absorbances of the mixtures were measured at 520 nm. Ascorbic acid was used as standard.

The antioxidant activities of the extract and fractions were evaluated by comparing their absorbencies with that of the negative control (0.5 mL of DPPH solution and 4.5 mL of methanol). The free radical scavenging activities were obtained using the relationship shown below:

 DPPH scavenging activity = 100 {(AC⁡−AS)/AC⁡}
 AC = Absorbance of negative control
 AS = Absorbance of sample.

### 2.6. Statistical Analysis

Graphical determination of IC_50_s and comparative presentations were done using SPSS version 17.

## 3. Results

### 3.1. Fractionation and Phytochemical Analysis

The distribution of phytocompounds and their yield in the crude precipitate, compound **1**, ethyl acetate, acetone, and methanol fractions is as shown in Table 1. Compound **1** was obtained in appreciable quantity. Due to the low yield of compound **2**, no further analysis was done on it. Preliminary phytochemical analysis of the two isolated compounds revealed that they are triterpenes.

### 3.2. DPPH Test

**Figure 1:**
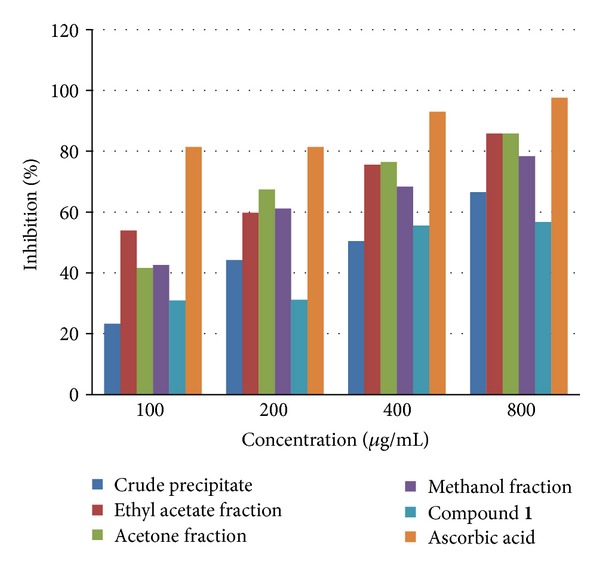
Free radical scavenging activity of crude precipitate, fractions, and compound **1** in comparison with ascorbic acid.

Graphical presentations of the percentage inhibitions of DPPH radical by the extract, crude precipitate, compound **1**, and fractions from *Alstonia boonei* are as shown in Figure 1 compared with ascorbic acid. The IC_50_ values (*μ*g/mL) were as shown in Table 2. The precipitate, fraction, and compound showed a dose response inhibition of DPPH radical. The order of activity is as follows: ethyl acetate fraction > acetone fraction > methanol fraction > compound **1** > crude precipitate.

## 4. Discussion

Several literature reports show that reduction in oxidative damage to DNA, proteins, and lipids enhances longevity and health span [11, 12]. Phytochemical analysis of the crude precipitate, fractions, and compound from the methanol root-bark extract of *A. boonei* showed the presence of phytochemicals which have been demonstrated to possess antioxidant properties such as alkaloids [13], steroids [14], saponins [15], triterpenes [16], flavonoids [17], tannins [18], and glycosides [19]. The presence of these phytocompounds may have contributed to the antioxidant activity exhibited by the fractions, precipitate, and isolated compound. DPPH method for evaluation of antioxidant properties of compounds and plant extracts is quick, reliable, and highly reproducible [20]. The inhibition of DPPH radical exhibited by the ethyl acetate, acetone, and methanol fractions could be explained by the presence of phenolic compounds in these fractions. The best-described property of most phenolic compounds is their capacity to act as antioxidants [21]. The antioxidant activity of phenolic compounds has been attributed to their high content of hydroxyl groups and their reducing potentials through electron −/H-donation. Phenolic compounds are known to concentrate in ethyl acetate fractions [22] and might have accounted for the high antioxidant activity exhibited by this fraction compared with other fractions, precipitate, and isolated compound. Also significant inhibition of DPPH radical by ethyl acetate fraction of *A. scholaris* belonging to the same family with *A. boonei* (Apocynaceae) has been documented and activity attributed to presence of phenolic compounds [23]. Steroids have good antioxidant activity acting mainly through membrane stabilization [24]; however, their poor solubility in polar reaction medium may hinder free interaction with DPPH radical in methanol solution. This may have contributed to the decreased DPPH inhibition exhibited by the crude precipitate and isolated compound.

## 5. Conclusion 

The establishment of the presence of antioxidant effect in the root-bark extracts of *A. boonei* goes further to validate our hypothesis that the numerous medicinal properties of the plant may not be unconnected to the presence of antioxidant activity. Structure elucidation of compound **1 **and the isolation and structure elucidation of the phenolic compounds present in ethyl acetate and acetone fractions are currently in process in our laboratory.

## Figures and Tables

**Table 1:** Phytochemical constituents and yields of crude precipitate, fractions, and compounds.

Extracts/fractions	Yield (% w/w)	Phytochemical constituents
Crude precipitate	0.91^a^	Steroids and triterpenes
Ethylacetate fraction	24.0^b^	Alkaloids, saponins, flavonoids, tannins, glycosides, and resins
Acetone fraction	20.0^b^	Saponins, flavonoids, tannins, and glycosides
Methanol fraction	50.0^b^	Alkaloids, saponins, flavonoids, tanins, and glycosides
Compound **1**	13.07^b^	Triterpenoid
Compound **2**	1.2^b^	Triterpenoid

^a^Yield calculated from 350 g of powdered root-bark.

^b^Yield calculated from 5 g of methanol extract.

**Table 2:** Free radical scavenging IC_50_ values of the fractions, compound **1**, and ascorbic acid.

Fractions/compound	IC_50_ (µg/mL)
Crude precipitate	364.39
Ethyl acetate fraction	54.25
Acetone fraction	121.79
Methanol fraction	141.67
Compound **1**	354.94
Ascorbic acid	<50
